# Heart rate variability and suicidal thoughts and behaviour: study protocol for a systematic review

**DOI:** 10.1186/s13643-025-02954-5

**Published:** 2025-10-15

**Authors:** Sonja Omlor, Norbert Scherbaum, Inken Höller, Thomas Forkmann

**Affiliations:** 1 LVR-University-Hospital Essen, Department of Psychiatry and Psychotherapy, Medical Faculty, University of Duisburg-Essen, Essen, Germany; 2 Center for Translational Neuro- and Behavioral Sciences, University of Duisburg-Essen, Essen, Germany; 3 Department of Clinical Psychology and Psychotherapy, Charlotte Fresenius Hochschule, Düsseldorf, Germany; 4 Department of Clinical Psychology and Psychotherapy, University of Duisburg-Essen, Essen, Germany

**Keywords:** Suicidal thoughts, Suicidal behaviour, Heart rate variability, Heart rate, Biomarker

## Abstract

**Background:**

As 700,000 people per year commit suicide worldwide, improvements in suicide prevention and the prediction of suicide attempts or suicide in clinical care are mandatory. This systematic review aims to examine heart rate (HR) and heart rate variability (HRV) as risk factors for the development of suicidal thoughts and behaviour in clinical and non-clinical populations.

**Methods and analysis:**

The systematic review will be conducted in accordance with the Preferred Reporting Items for Systematic Review and Meta-Analysis Protocols (PRISMA). We will retrieve relevant literatures across the following databases: PubMed, PsycINFO, Cochrane Library and Web of Science. Additionally, we will manually search the reference lists of all relevant articles. If studies are published or translated in English and measure HRV or HR and suicidal thoughts or behaviour, they will be included. Two reviewers will independently complete the article selection, data extraction and risk of bias ratings. A third reviewer will resolve disagreements. Tabular and narrative synthesis will be done accordingly and a risk of bias assessment will be conducted by the QUIPS (Quality In Prognosis) tool.

**Discussion:**

This systematic review will present evidence from which conclusions can be made regarding the relationship between HRV and HR and suicidal thoughts and behaviour. By identifying risk factors associated with suicidality and differentiating between factors for suicidal thoughts versus behaviour, this review will potentially contribute theoretically to our understanding of the causal factors involved in influencing increasing levels of suicidality.

**Ethics and dissemination:**

This is a systematic review of published literature and thereby ethical approval was not sought. Results will be disseminated through conferences and publications in relevant peer-reviewed journals.

**Strength and limitations of this study:**

The results will have important implications for risk prediction for suicidal thoughts and behaviour. The protocol follows the PRISMA guidelines (Page et al.,BMJ 372:n71,2021). Thus, two reviewers will perform data extraction and risk of bias evaluation separately. We will exclude non-English translated or published studies, which might be a limitation and bias against non-English-speaking countries.

**Systematic review registration:**

PROSPERO registration number: CRD42023460068

## Introduction

As 700,000 people per year commit suicide worldwide [1], improvements in our understanding of risk factors for suicidal thoughts and behaviour are mandatory. Even though single risk factors such as male gender, advanced age, social isolation, depressive and psychotic illness, substance use and insomnia are well-studied, the prediction of suicide attempts or suicides remains imprecise. In a comprehensive meta-analysis, Franklin et al. [2] investigated potential risk factors for suicidal attempts and suicides of the past 50 years of research and concluded that prediction of suicidality did not substantially improve. Here, the consideration of proximal instead of solely distal risk factors was proposed to be promising. In epidemiology, risk factors are divided in proximal and distal risk factors. Proximal factors such as job loss describe temporally close risk factors for suicidality, whereas distal factors such as mental disorder or childhood abuse describe temporally distant risk factors of suicidality. Two highly discussed potential proximal risk factors for suicidal thoughts and behaviour are heart rate (HR) and heart rate variability (HRV) [3]. HR is discussed as a physiological indicator for arousal [4]. Some studies could find a positive association between completed suicide and increased HR [5, 6], but it is unclear if HR systematically predicts suicide ideation and behaviour. On the other hand, reduced HRV is discussed as a stress indicator for autonomic nervous system imbalance [7]. HRV has been investigated in two ways, the resting levels of heart rate variability and the reactivity of HRV [8]. In particular, the HRV reactivity indicates acute changes in self-regulation and state mood in the physiological response compared to rest [9] and might thus be a proximal risk factor. While in some studies a negative association between HRV and suicidal thoughts could be found [3, 10–12], other studies reported no significant association between HRV and suicidal thoughts [13].

The neurovisceral integration model by Thayer and Lane [14] might be a reasonable framework to describe the relation of HR and HRV on the one side and suicidal ideation and behaviour on the other side. The neurovisceral integration theory supposes that HRV is associated with the inhibition capability of the prefrontal cortex on subcortical structures. In suicidal patients, the inhibition capability is reduced [15]. Furthermore, according to Chang et al. [16], a higher intensity in suicidal thoughts was associated with a reduced HRV. Jelinek and Khandoker [12] report that HRV biofeedback training could reduce suicidal thoughts in depressive patients. Michael and Kaur [17] discuss in their review on the brain-heart connection that there is emerging evidence that rTMS treatment of the prefrontal cortex leads to reduced HR and increased HRV in depressive patients. Schiweck et al. [18] report in their systematic review that the majority of studies find hyperreactivity during stress in depressive patients shown by lower fluctuations in heart rate and heart rate variability in the high-frequency band. Even though this review suggests that there is a negative association between HRV and depression, the relationship between HRV and suicidal ideation and behaviour as correlate of depression remains unclear.

Even though HR and HRV are supposed to be rather proximal risk factors of suicidal ideation and behaviour, it is unclear how close to the emergence of suicidal thoughts and behaviour changes in HR and HRV might occur. Two theoretical models attempt to explain the temporal development of suicidal thoughts and behaviour: the Integrative Motivational-Volitional model (IMV) [19] and the Fluid Vulnerability theory [20]. The IMV model divides the suicidal process in a motivational and volitional phase and proposes that moderating factors such as social problem solving, impulsivity or physical pain sensitivity promote or inhibit the suicidal development. Here HRV and HR could be supposed to be additional moderator variables in an early stage of the process, the motivational phase, and promote the emergence of entrapment and then suicidal thoughts. On the other hand, HR and HRV could also perform as potential moderator variables in a later stage of the process, the volitional phase, and promote the transition from suicidal ideation to behaviour. According to the IMV model, suicidal thoughts are a prerequisite for suicidal behaviour. We are interested if this literature search finds HR and HRV associations in early or late stages of suicidal crisis. We would like to understand if those two potential physiological biomarkers, HR and HRV, are altered rather in the motivational or volitional phase of the IMV model.

Another theory, the Fluid Vulnerability theory [20] distinguishes between static and dynamic aspects of suicidal risk. HRV reactivity and HR might be conceptualized as rather dynamic aspects of suicidal risks. This theory proposes non-linear changes in suicidal thoughts and behaviour. We are interested if an altered HRV or HR is associated with those short-term suicidal thoughts and behaviour changes.

### Why is this review important?

Previous reviews have focused generally on HRV and stress reaction [21] or HRV and depression [18] or HRV and suicidal behaviour [7], or HRV and cognitive function [22]. We, instead, are interested in suicidal thoughts and behaviour and its temporal development. We are particularly interested if the literature proposes that HR and HRV are associated with both variables, suicidal thoughts and behaviour, or only one of them. Furthermore, this review aims to investigate if both, HR and HRV, or one of them is altered in an early or later stage of the suicidal crisis. We are as well interested in investigations of HR and HRV in populations without mental disorders such as students or other non-clinical populations, as the DSM-5 [23] defines suicidality as own diagnostic category instead of a symptom of a mental disorder such as depression or borderline personality disorder. Thus, this review searches for suicidal ideation and behaviour transdiagnostically, in clinical and non-clinical populations.

In contrast to the systematic review by Kang et al. [24] that addresses similar research questions, our review brings up a different focus on the topic regarding six main differences. First, we are interested in the differentiation between suicidal thoughts and behaviour instead of the ambiguous generic term suicidality. Second, ecological momentary assessments (EMA) with wearable devices such as smart watches or smart phones are being increasingly used to measure psychological symptoms such as suicidal thoughts and physiological symptoms such as HR [25]. Thus, we are not only interested in baseline HR and HRV measurements or single suicidality measurements, but also in its temporal development and thereby in continuously collected data by, e.g. EMA study designs. This coincides with the request in Kang et al. [24] that the bidirectional relationship between HRV and suicide needs to be understood. Third, in our review, we would like to differentiate recordings of electric activity by ECG measurements and measurement based on plethysmography by wearable devices such as fitness watches for HR and HRV. Kang et al. [24] found in total 11 HR/HRV studies with only ECG measurements, but none with wearable devices. We instead expect measurements by wearable devices to be increasingly used in the last years such as in the study by Sheridan et al. [3] so that an updated review of the literature is warranted. Fourth, accordingly, we expect several studies being published in the last 4 years after publication of the review by Kang et al. [24], as the investigation of warning signals and proximal instead of distal risk factors of suicidal ideation and behaviour is of higher interest in the literature now, since Franklin et al. [2] emphasized their importance. Fifth, Kang et al. [24] searched in only two databases (PubMed, EMBASE), while we will search for eligible papers in four databases (PubMed, PsycINFO, Web of Science and the Cochrane database). Sixth, we will offer a risk of bias assessment, which is missing in the systematic review of Kang et al. [24].

## Methods

The review will be conducted according to the PRISMA (Preferred Reporting Items for Systematic Review and Meta-Analysis Protocols) guidelines for reporting systematic reviews [26].

### Research question

We will apply the adapted version of the PICO (P = Patients, I = Intervention, C = Comparison, O = Outcomes) method for systematic reviews of association, the PEO method [27], which defines the major components of this systematic review. In general, these components are as follows: Population (P; types of participants), Exposure of interest (E; independent variable), Outcome or response (O; dependent variable). With regard to our systematic review, the PEO components are defined as follows:

- P: Clinical and non-clinical populations, who report suicidal thoughts or behaviour. Any gender and ethnic background will be included.
- E: The prognostic factor or independent variable is the HR or HRV of people with suicidal thoughts or behaviour.
- O: Increased risk of suicidal thoughts and behaviour.

Thus, the review aims to address the following research questions:

1. Which role does HR and HRV play for the prediction of suicidal thoughts and behaviour?
2. Is there an association between HR and the intensity or frequency of suicidal thoughts or behaviour?
3. Is there an association between HRV and the intensity and frequency of suicidal thoughts or behaviour?

### Inclusion criteria

We will include (1) studies that measured HRV or HR and (2) measured suicidal thoughts or behaviour and (3) report the association between HR/HRV and suicidal thoughts or behaviour in (4) clinical and non-clinical populations, (5) quantitative research studies, (6) experimental, non-experimental and hybrid study designs (longitudinal, cross-sectional, cohort studies and intervention studies), (7) published in or translated to English and (8) published in a peer-reviewed journal. There will be no restriction on year of publication.

### Exclusion criteria

Studies (1) that did not measure the intensity or frequency of suicidal thoughts or behaviour or (2) that did not quantitatively measure HR or HRV will be excluded. (3) We will also exclude non-peer-reviewed (4) and non-English articles (5). We will exclude studies that do not investigate the association between HR/HRV and suicidal thoughts and behaviour (6). We will exclude studies that are not human-based studies (7). We will exclude studies with no experimental, non-experimental or hybrid study design (8). We will exclude articles that are case reports, reviews or meta-analyses (9). We will exclude study protocols (10) and duplicates (11).

### Search strategy

We will apply the adapted PICO approach [28], which we will name the PEO approach [27], in order to define the major components of this systematic review, with P for population (‘suicid*’) with E for Exposure of interest (‘heart rate variability’ OR ‘heart rate’ OR ‘HRV’ OR ‘Cardiac Vagal Control’ OR ‘Cardiac Vagal Tone’ OR ‘Autonomic Nervous System’ OR ‘Cardio-Vascular Reactivity’) and O for outcome (‘suicid*’). The search strategy will comprise a combination of keywords or respectively MeSH terms (in the PubMed database), Boolean operators and truncation. The following PRISMA compliant search strategy will be applied in the databases: Suicid* AND (‘respiratory sinus arrhythmia’ OR ‘heart rate variability’ OR ‘heart rate’ OR ‘HRV’ OR ‘Cardiac Vagal Control’ OR ‘Cardiac Vagal Tone’ OR ‘Autonomic Nervous System’ OR ‘Cardio-Vascular Reactivity’). We will also apply specific filters such as ‘peer-reviewed journals’ and ‘all years’ as, e.g. the database PsycINFO offers. The search will be undertaken using the PubMed database, the PsycINFO database, the Web of Science database and the Cochrane database. Reference lists of full-text articles included in the screening will be manually searched for potential literature.

### Study selection

Studies of the database search and studies picked by hand searching of the reference lists will be screened. First, studies will be selected by screening the title and abstract information. In a second step, the full texts of those studies included after abstract screening will be read to finally decide about study inclusion. The number of duplicates resulting from the searches in the different databases will be reported and duplicates will be removed. Two authors (TF, SO) will independently extract data from the included studies. The authors will independently review titles, abstracts and full texts of each study and indicate on a study eligibility form if the study should be included, excluded, or if they are undecided. If there is any disagreement, we will discuss with a third member (IH) of the review team and describe decisions. If necessary, we will contact authors of studies for clarification.

### Data extraction

The following information will be extracted: (1) author(s) and year of publication, (2) study design (prospective, retrospective or cross-sectional), (3) characteristics of participants (including sample size, age, gender, mental disorder versus healthy people, with/without psychopharmacological medication). (4) For higher transparency and visualization, we will supply a column, which indicates the HR and/or HRV (yes/no) measurement and suicidal ideation and/or suicidal behaviour (yes/no) measurement for each included study. (5) We will in addition extract the type of HRV and HR measure such as ECG or photoplethysmography with wearable devices (including measurements in the time domain (e.g. RMSSD (root mean square of successive RR interval differences)) or frequency domain (e.g. HF peak HRV (peak frequency of the high-frequency band (0.15–0.4 Hz))) and the recording duration (e.g. 24 h, short-term (~5 min) or ultra-short-term (<5 min)). (6) We will also extract the reported outcome measure of suicidality (measurements of suicidal thoughts or behaviour in frequency or intensity and the applied self-report questionnaire such as BSS or interview such as SITBI) and the (7) size and direction of the identified relationship. Two reviewers will independently use this structured form for data extraction.

### Risk of bias (quality) assessment

For all included articles, two reviewers will independently assess the risk of bias according to the GRADE tool [29], which offers categories to consider when drawing conclusions about causality. This ought to mitigate selection bias and publication bias. As our systematic review topic is novel and studies in this field of research have rather recently been published, we expect that there might be a publication bias towards significant findings and there might be the file drawer problem. In research topics that have been studied in the field for much longer, more replication studies might exist and less publication bias might be a problem. For that reason, we decided to use a further risk of bias tool to address this problem of assumed higher publication bias in our new research topic of interest. As we are interested in prognosis questions, we additionally apply a prognosis-specific risk of bias tool, the Quality In Prognosis Studies (QUIPS) tool [30]. Here, two reviewers will independently assess the risk of bias across six domains. The Joanna Briggs Institute’s approach to conduct systematic reviews of association and the Cochrane Prognosis Methods Group recommend the use of the QUIPS tool [27, 31] in order to assess the risk of bias in prognostic factor studies. This tool is developed to evaluate validity and bias in studies of prognostic factors. It considers the following six important areas: (1) study participation, (2) study attrition, (3) prognostic factor measurement, (4) confounding measurement, (5) outcome measurement as well as (6) statistical analysis and reporting. For all studies included in the review, two reviewers will independently rate the three to seven prompting items of each assessment area on a four-grade scale (yes, partial, no, unsure). To evaluate the risk of bias, each reviewer makes an overall, conclusive decision within each domain on the basis of the item ratings. Disagreements in the overall judgements (low, moderate, high risk of bias) will be resolved by consensus with a third reviewer. Ratings of bias for each study will be classified as low, moderate or high based on standardized methodology.

### Strategy for data synthesis and analysis

#### Tabular synthesis of data

We will provide a tabular synthesis of the findings from the included studies, structured around target population (healthy people/people with acute mental disorder/people with remitted mental disorder/people with and without psychopharmacological medication) and around study design (prospective, retrospective, cross-sectional). In addition, we will report in the tabular summary the findings regarding (1) age group, (2) objectives, (3) exposure type (heart rate or heart rate variability), (4) outcome type (suicidal thoughts or behaviour), (5) exposure measurement method (e.g. wearable device or ECG) and outcome measurement method (e.g. interview or questionnaire) and (6) scientific quality.

Data will be expressed as descriptive statistics only. Heterogeneity will be qualitatively assessed in terms of study design, population, key result (regarding association between HR(V) and suicidal thoughts and behaviour) and measurement methods. The relationship between suicidal thoughts and behaviour and heart rate and heart rate variability will be discussed.

According to our inclusion criteria, we assume the found studies to be heterogeneous. Our assumption is based on our inclusion of prospective as well as cross-sectional and retrospective study designs and included various measurement methods in terms of suicidality (interview, different types of questionnaires) and in terms of heart rate and heart rate variability (wearable devices, ECG). As we expect the included studies to be inhomogeneous in a clinical and methodological point of view, we do not plan to calculate and pool effect sizes, such as in a random-effect meta-analysis. As we expect conditions to impede meta-analyses, we will instead narratively present statistical characteristic values such as *M*, *SD*, *N* and the calculated statistical tests (*t*-test, correlation, ANOVA, etc.) of each included study and present them in tabular form. We will narratively describe study results grouped by HRV index reported and characteristics of the study population. Patterns of association between HRV and suicidal ideation and behaviour, potential mechanisms of this association and important themes will be identified and discussed. After applying the GRADE and QUIPS tool, we will report narratively the studies of low risk of bias.

## Discussion

This review will synthesize all available data on the relationship between HR/HRV and suicidal thoughts and behaviour. The results will show if there is a consistent positive or negative association between HR/HRV and suicidal thoughts and behaviour or if no directed association is found or if HR/HRV indices fluctuate with the presence of suicidal thoughts and behaviour. In the sequel, hypotheses can be formed if alterations in HR/HRV indices are non-linear. Here, the Fluid Vulnerability theory model proposes alterations of risk factors within the suicidal crisis to be non-linear [32]. The findings of this systematic review will contribute to our understanding of HR and HRV as a potential physiological risk factor for the development of suicide pathology regarding suicidal thoughts and behaviour. Within the IMV model [19], we can interpret results either that HR/HRV is a moderator variable of the motivational or volitional phase: If results indicate that suicide ideation only is associated with HR/HRV indices, they could be interpreted as moderator variable of the motivational phase. If the physiological markers are rather linked to suicide behaviour only, the heart rate (variability) might rather serve as moderator variable of the volitional phase. From the results of this review, we might deduce important strategies of risk assessment and diagnostical approaches in suicidal management which might include the assessment of physiological markers such as HR/HRV. In addition, the results might imply a need to integrate HR/HRV screening in the treatment of suicidal patients. This review should enhance the intervention efforts, either by revealing physiological alterations that are side effects of the development of suicidality, or by directly informing about probable targets for psychiatric and psychotherapeutic interventions. There is first evidence that HRV parameters could inform about the response to antidepressant treatment in depressive patients [33]. Thus, probable HR/HRV alterations in the depressive subgroup of suicidal patients is conceivable. However, it is also possible that the response to antidepressant treatment in the suicidal depressive subgroup is not associated with HR/HRV alterations. In a further step, such findings on HR/HRV alteration and the association to medicated or unmedicated suicidal patients could be useful to refine intervention strategies and to better address suitable interventions to patients’ subgroups (e.g. with high, low HRV). As HRV is discussed as stress indicator for autonomic nervous system imbalance [7], intervention targeting the autonomous nervous system could be promising in the treatment of suicidal patients. HRV interventions that address the vagus nerve system, such as progressive muscle relaxation (PMR) [34] or sports [35], have been reported to lower depressive symptoms in depressive patients. As suicidality is a probable symptom of major depression, it is conceivable that also suicidal symptoms such as suicidal thoughts and behaviour could be ameliorated after interventions that impact the vagus nerve system, such as PMR or sports intervention. With the aid of this review, it could be examined if HR/HRV alterations are consistently reported in suicidal patients and could be a target for interventions. If we will find consistent HR/HRV alterations and associations with suicidality, the psychological marker of HR/HRV could be helpful to quantify an improvement in the intensity and frequency of suicidal thoughts and behaviour. In general, the review results have the potential to inform and expand current theoretical models of the development of suicidal thoughts and behaviour such as the IMV model or the Fluid Vulnerability model [32, 36]. These findings, as well as the appraisal of the status of the literature, might be of use to clinicians, in identifying individuals at increased risk of suicide. Furthermore, they may impact prevention tools with important implications for public health policy. The heterogeneity between studies regarding study design, the heterogeneity of the reported indices of HRV (e.g. RMSSD (i.e. root mean square of successive RR interval differences) or HF peak HRV (i.e. peak frequency of the high-frequency band (0.15–0.4 Hz)), and the measurement methods of suicidal ideation and behaviour as well as the heterogeneity in the sample size of each study and the general low base rate of suicidal behaviour might be a limitation of the review and might impact the generalizability of results. The restriction to studies published in or translated to English might introduce risk of language or publication bias as some potentially relevant non-English studies could be excluded from analyses. Although this appears to be common practice as many systematic reviews rely on research published in or translated to English, this practice may contribute to perpetuating marginalizing regional journals or research from non-English-speaking countries. Thus, due to practical reasons, we will not include non-English literature. However, this issue will be discussed broadly as a potentially important limitation in the final systematic review.

## Bibliometric and software considerations

The web-based tool ‘nestedknowledge’ will be used to store, manage and screen the results of the searches.

## Data Availability

Not applicable.

## References

[1] Preventing suicide: a global imperative. [cited 2023 Sep 21]. Available from: https://www.who.int/publications/i/item/9789241564779.

[2] Franklin JC, Ribeiro JD, Fox KR, Bentley KH, Kleiman EM, Huang X, et al. Risk factors for suicidal thoughts and behaviors: a meta-analysis of 50 years of research. Psychol Bull. 2017;143(2):187–232. 27841450 10.1037/bul0000084

[3] Sheridan DC, Baker S, Dehart R, Lin A, Hansen M, Tereshchenko LG, et al. Heart rate variability and its ability to detect worsening suicidality in adolescents: a pilot trial of wearable technology. Psychiatry Investig. 2021;18(10):928–35. 34555890 10.30773/pi.2021.0057 PMC8542751

[4] Thayer RE. Activation states as assessed by verbal report and four psychophysiological variables. Psychophysiology. 1970;7(1):86–94. 5492738 10.1111/j.1469-8986.1970.tb02278.x

[5] Lemogne C, Thomas F, Consoli SM, Pannier B, Jégo B, Danchin N. Heart rate and completed suicide: evidence from the IPC cohort study. Psychosom Med. 2011;73(9):731–6. 22021462 10.1097/PSY.0b013e3182365dc7

[6] Chang SS, Bjørngaard JH, Tsai MK, Bjerkeset O, Wen CP, Yip PSF, et al. Heart rate and suicide: findings from two cohorts of 533 000 Taiwanese and 75 000 Norwegian adults. Acta Psychiatr Scand. 2016;133(4):277–88. 26493376 10.1111/acps.12513

[7] McCall WV, Rosenquist PB, Miller BJ. Development of autonomic nervous system assays as point-of-care tests to supplement clinical judgment in risk assessment for suicidal behavior: a review. Curr Psychiatry Rep. 2022;24(1):11–21. 35076889 10.1007/s11920-022-01315-6

[8] Hamilton JL, Alloy LB. Atypical reactivity of heart rate variability to stress and depression across development: systematic review of the literature and directions for future research. Clin Psychol Rev. 2016;50:67–79. 27697746 10.1016/j.cpr.2016.09.003 PMC5233715

[9] Beauchaine T. Vagal tone, development, and Gray’s motivational theory: toward an integrated model of autonomic nervous system functioning in psychopathology. Dev Psychopathol. 2001;13(2):183–214. 11393643 10.1017/s0954579401002012

[10] Forkmann T, Meessen J, Teismann T, Sütterlin S, Gauggel S, Mainz V. Resting vagal tone is negatively associated with suicide ideation. J Affect Disord. 2016;194:30–2. 26802504 10.1016/j.jad.2016.01.032

[11] Chang HA, Chang CC, Chen CL, Kuo TBJ, Lu RB, Huang SY. Major depression is associated with cardiac autonomic dysregulation. Acta Neuropsychiatr. 2012;24(6):318–27. 25287173 10.1111/j.1601-5215.2011.00647.x

[12] Jelinek HF, Khandoker AH. Reducing suicidal ideation by biofeedback-guided respiration – heart rate coherence. Digital Psychiatry. 2020;3(1):1–11.

[13] Liang CS, Lee JF, Chen CC, Chang YC. Reactive heart rate variability in male patients with first-episode major depressive disorder. Prog Neuropsychopharmacol Biol Psychiatry. 2015;56:52–7. 25149628 10.1016/j.pnpbp.2014.08.004

[14] Thayer JF, Lane RD. A model of neurovisceral integration in emotion regulation and dysregulation. J Affect Disord. 2000;61(3):201–16. 11163422 10.1016/s0165-0327(00)00338-4

[15] Gujral S, Dombrovski AY, Butters M, Clark L, Reynolds CF, Szanto K. Impaired executive function in contemplated and attempted suicide in late life. Am J Geriatr Psychiatry. 2014;22(8):811–9. 23567385 10.1016/j.jagp.2013.01.025 PMC3516623

[16] Chang HA, Chang CC, Chen CL, Kuo TBJ, Lu RB, Huang SY. Heart rate variability in patients with fully remitted major depressive disorder. Acta Neuropsychiatr. 2013;25(1):33–42. 26953072 10.1111/j.1601-5215.2012.00658.x

[17] Michael JA, Kaur M. The heart-brain connection in depression: can it inform a personalised approach for repetitive transcranial magnetic stimulation (rTMS) treatment? Neurosci Biobehav Rev. 2021;127:136–43. 33891972 10.1016/j.neubiorev.2021.04.016

[18] Schiweck C, Piette D, Berckmans D, Claes S, Vrieze E. Heart rate and high frequency heart rate variability during stress as biomarker for clinical depression. A systematic review. Psychol Med. 2019;49(2):200–11. 30134999 10.1017/S0033291718001988

[19] O'Connor RC, Kirtley OJ. The integrated motivational-volitional model of suicidal behaviour. Philos Trans R Soc Lond B Biol Sci. 2018;373(1754):20170268. 10.1098/rstb.2017.0268. PMID: 30012735; PMCID: PMC6053985. 10.1098/rstb.2017.0268 PMC6053985 30012735

[20] Rudd MD. Fluid vulnerability theory: a cognitive approach to understanding the process of acute and chronic suicide risk. Cogn Suicide Theory Res Ther. 2007;13:355–68.

[21] Kim HG, Cheon EJ, Bai DS, Lee YH, Koo BH. Stress and heart rate variability: a meta-analysis and review of the literature. Psychiatry Investig. 2018;15(3):235–45. 29486547 10.30773/pi.2017.08.17 PMC5900369

[22] Forte G, Favieri F, Casagrande M. Heart rate variability and cognitive function: a systematic review. Front Neurosci. 2019;13:710. 31354419 10.3389/fnins.2019.00710 PMC6637318

[23] American Psychiatric Association. Diagnostic and statistical manual of mental disorders. Diagnostic and statistical manual of mental disorders. 2013.

[24] Kang GE, Patriquin MA, Nguyen H, Oh H, Rufino KA, Storch EA, et al. Objective measurement of sleep, heart rate, heart rate variability, and physical activity in suicidality: a systematic review. J Affect Disord. 2020;273:318–27. 32421619 10.1016/j.jad.2020.03.096 PMC7306422

[25] Wrzus C, Neubauer AB. Ecological momentary assessment: a meta-analysis on designs, samples, and compliance across research fields. Assessment. 2023;30(3):825–46. 35016567 10.1177/10731911211067538 PMC9999286

[26] Page MJ, McKenzie JE, Bossuyt PM, Boutron I, Hoffmann TC, Mulrow CD, et al. The PRISMA 2020 statement: an updated guideline for reporting systematic reviews. BMJ. 2021;372:n71. 33782057 10.1136/bmj.n71 PMC8005924

[27] Moola S, Munn Z, Sears K, Sfetcu R, Currie M, Lisy K, et al. Conducting systematic reviews of association (etiology): the Joanna Briggs Institute’s approach. Int J Evid Based Healthc. 2015;13(3):163–9. 26262566 10.1097/XEB.0000000000000064

[28] Liberati A, Altman DG, Tetzlaff J, Mulrow C, Gøtzsche PC, Ioannidis JPA, et al. The PRISMA statement for reporting systematic reviews and meta-analyses of studies that evaluate health care interventions: explanation and elaboration. PLoS Med. 2009;6(7):e1000100. 19621070 10.1371/journal.pmed.1000100 PMC2707010

[29] Shimonovich M, Pearce A, Thomson H, Keyes K, Katikireddi SV. Assessing causality in epidemiology: revisiting Bradford Hill to incorporate developments in causal thinking. Eur J Epidemiol. 2021;36(9):873–87. 33324996 10.1007/s10654-020-00703-7 PMC8206235

[30] Hayden JA, van der Windt DA, Cartwright JL, Côté P, Bombardier C. Assessing bias in studies of prognostic factors. Ann Intern Med. 2013;158(4):280–6. Avaliable from: https://www.acpjournals.org/doi/abs/10.7326/0003-4819-158-4-201302190-00009. 23420236 10.7326/0003-4819-158-4-201302190-00009

[31] Group CCPM. The Cochrane Collaboration Prognosis Methods Group. Review tools. 2018.

[32] Rudd MD. Fluid vulnerability theory: a cognitive approach to understanding the process of acute and chronic suicide risk. Cogn Suicide Theory Res Ther. 2007;13:355–68.

[33] Novello S, Hartmann R, Schmidt FM, Sander C, Hegerl U. Heart rate variability as indicator of clinical state in depression. 2019. Available from: www.frontiersin.org. 10.3389/fpsyt.2018.00735 PMC6344433 30705641

[34] Muhammad Khir S, Wan Mohd Yunus WMA, Mahmud N, Wang R, Panatik SA, Mohd Sukor MS, et al. Efficacy of progressive muscle relaxation in adults for stress, anxiety, and depression: a systematic review. Psychol Res Behav Manag. 2024;17:345–65. 38322293 10.2147/PRBM.S437277 PMC10844009

[35] Schuch FB, Vancampfort D, Firth J, Rosenbaum S, Ward PB, Silva ES, et al. Physical activity and incident depression: a meta-analysis of prospective cohort studies. Am J Psychiatry. 2018;175(7):631–48. 10.1176/appi.ajp.2018.17111194. 29690792 10.1176/appi.ajp.2018.17111194

[36] Branley-Bell D, O’Connor DB, Green JA, Ferguson E, O’Carroll RE, O’Connor RC. Distinguishing suicide ideation from suicide attempts: further test of the integrated motivational-volitional model of suicidal behaviour. J Psychiatr Res. 2019;1(117):100–7. 10.1016/j.jpsychires.2019.07.007 31376620

